# Correlation analyses of clinical and molecular findings identify candidate biological pathways in systemic juvenile idiopathic arthritis

**DOI:** 10.1186/1741-7015-10-125

**Published:** 2012-10-23

**Authors:** Xuefeng B Ling, Claudia Macaubas, Heather C Alexander, Qiaojun Wen, Edward Chen, Sihua Peng, Yue Sun, Chetan Deshpande, Kuang-Hung Pan, Richard Lin, Chih-Jian Lih, Sheng-Yung P Chang, Tzielan Lee, Christy Sandborg, Ann B Begovich, Stanley N Cohen, Elizabeth D Mellins

**Affiliations:** 1 Department of Surgery, Stanford University, Stanford, CA 94305, USA; 2 Program in Immunology, Department of Pediatrics, Stanford University, Stanford, CA 94305, USA; 3 Celera Corporation, Alameda, CA 94502, USA; 4 Department of Genetics, Stanford, CA 94305, USA; 5 Division of Pediatric Rheumatology, Department of Pediatrics, Stanford University, Stanford, CA 94305, USA

**Keywords:** Arthritis, Inflammation, Juvenile idiopathic arthritis (JIA), Systemic JIA, Polyarticular JIA, Transcriptional analysis

## Abstract

**Background:**

Clinicians have long appreciated the distinct phenotype of systemic juvenile idiopathic arthritis (SJIA) compared to polyarticular juvenile idiopathic arthritis (POLY). We hypothesized that gene expression profiles of peripheral blood mononuclear cells (PBMC) from children with each disease would reveal distinct biological pathways when analyzed for significant associations with elevations in two markers of JIA activity, erythrocyte sedimentation rate (ESR) and number of affected joints (joint count, JC).

**Methods:**

PBMC RNA from SJIA and POLY patients was profiled by kinetic PCR to analyze expression of 181 genes, selected for relevance to immune response pathways. Pearson correlation and Student's *t*-test analyses were performed to identify transcripts significantly associated with clinical parameters (ESR and JC) in SJIA or POLY samples. These transcripts were used to find related biological pathways.

**Results:**

Combining Pearson and *t*-test analyses, we found 91 ESR-related and 92 JC-related genes in SJIA. For POLY, 20 ESR-related and 0 JC-related genes were found. Using Ingenuity Systems Pathways Analysis, we identified SJIA ESR-related and JC-related pathways. The two sets of pathways are strongly correlated. In contrast, there is a weaker correlation between SJIA and POLY ESR-related pathways. Notably, distinct biological processes were found to correlate with JC in samples from the earlier systemic plus arthritic phase (SAF) of SJIA compared to samples from the later arthritis-predominant phase (AF). Within the SJIA SAF group, IL-10 expression was related to JC, whereas lack of IL-4 appeared to characterize the chronic arthritis (AF) subgroup.

**Conclusions:**

The strong correlation between pathways implicated in elevations of both ESR and JC in SJIA argues that the systemic and arthritic components of the disease are related mechanistically. Inflammatory pathways in SJIA are distinct from those in POLY course JIA, consistent with differences in clinically appreciated target organs. The limited number of ESR-related SJIA genes that also are associated with elevations of ESR in POLY implies that the SJIA associations are specific for SJIA, at least to some degree. The distinct pathways associated with arthritis in early and late SJIA raise the possibility that different immunobiology underlies arthritis over the course of SJIA.

## Background

Systemic juvenile idiopathic arthritis (SJIA) is currently classified as a subtype of juvenile idiopathic arthritis [1], and is characterized by a combination of arthritis and systemic inflammation, including fever, rash and serositis. SJIA has distinct demographic characteristics compared to other JIA subtypes, including onset throughout childhood and lack of gender preference. At clinical presentation, SJIA may resemble other diseases in children, including viral infection and Kawasaki disease [2-4]. The outcome in SJIA is variable, with close to half of children having a monocyclic course, less than 10% having an intermittent course, and over half having a persistent course [5,6], the latter often dominated by chronic arthritis. An adult form of SJIA is called Adult Onset Still Disease (AOSD) and occurs rarely [7].

There are also unique immunophenotypic features in SJIA compared to other JIA subtypes, such as the lack of human leukocyte antigen (HLA) class II allele association, low or absent autoantibodies (specifically, antinuclear antibodies, rheumatoid factor or anti-CCP antibodies [8]), a tendency toward monocytosis [9,10], high levels of IL-18 [11,12] and natural killer cell abnormalities in at least a subset of patients [13]. These immunologic features, together with the therapeutic efficacy of inhibitors of IL-1 or IL-6 in SJIA and AOSD, suggest that these diseases might be best classified as autoinflammatory rather than autoimmune [14-17].

Despite our knowledge of some important immunological characteristics of active SJIA, the pathogenesis of SJIA remains unknown. One of the unanswered questions is whether independent biological processes underlie the systemic symptoms and the arthritis. Evidence from clinical studies shows that earlier in the disease, IL-1 inhibitors (and perhaps also IL-6 blockade) are efficacious, especially against systemic symptoms, but at a later stage, where arthritis may predominate, patients may develop resistance to these therapies [18-20]. These findings suggest that distinct biological processes may be associated with different manifestations and/or different stages of the disease.

Transcriptional profiling of peripheral blood cells has been a useful approach for identifying biological pathways involved in SJIA and other complex diseases, such as polyarticular JIA (POLY), rheumatoid arthritis (RA), systemic lupus erythematosus and Kawasaki disease [21-24]. Previous studies of SJIA using microarray analyses have revealed transcriptional signatures in peripheral blood associated with active disease and with patient subsets [25-29].

We hypothesized that distinct gene expression patterns may be associated with individual clinical parameters used as measures of the systemic inflammation and the arthritis. We analyzed expression in peripheral blood mononuclear cells (PBMC) of a panel of inflammation-associated genes to determine patterns associated with elevations in two markers of disease activity in JIA, erythrocyte sedimentation rate (ESR) and number of active joints (joint count, JC). ESR is a marker of inflammation that is elevated in association with systemic as well as organ-specific inflammation, including arthritis [30]. Active joints are defined as joints with non-bony swelling or limited range of motion, with either tenderness or pain on motion; we chose active joint count as a marker of arthritis.

We asked if common or unique expression profiles are associated with ESR and JC in SJIA. In order to assess the specificity of our results for SJIA, we also asked whether the expression of the panel of tested genes differed in SJIA patients compared to patients with polyarticular course JIA (POLY), which is characterized by chronic polyarthritis. We then analyzed if JC associated genes differ during the early and late phase of SJIA. Based on the gene expression patterns, we identified candidate biological pathways associated with the systemic and arthritis components of SJIA.

## Methods

### Subject population and clinical data collection

The study was approved by the Stanford University Administrative Panel on Human Subjects in Medical Research (protocol ID 13932). Informed consent was obtained from patients or parents or guardians before blood sample collection. Venous blood samples from all subjects were treated anonymously throughout the analysis. All JIA patients were followed at the Pediatric Rheumatology Clinic at Lucile Packard Children's Hospital. SJIA and POLY patients met amended ILAR criteria for diagnosis [1]. Thirty-one SJIA and 18 POLY individual patients participated in this study. A total of 46 SJIA samples (22 Flare and 24 Quiescence samples), and 25 POLY samples (17 Flare and 8 Quiescence samples) were analyzed. Some patients, (SJIA n = 15; POLY n = 7) contributed samples during both flare and quiescent disease states. Twelve POLY patients were rheumatoid factor (RF) negative, and six were RF positive. All samples were classified as flare (F) or quiescence (Q) based on a scheme we developed for this and other studies of JIA [10,31,32] (Tables 1, 2 and 3 and [33,34]). SJIA flare samples had a systemic score of ≥ 1 and/or an arthritis score of ≥ B (≥ 5 active joints). POLY flare samples had an arthritis score of ≥ 1 (≥ 1 to 10 active joints). Arthritis severity is scored differently for SJIA and POLY patients, because the patterns of joint involvement generally are different between the two groups [34], with the exception that some SJIA patients develop POLY-like arthritis with symmetric, small joint involvement. The arthritis scoring system is based on frequency analyses of numbers of active joints in early active SJIA [34] and in active POLY [Sandborg C, frequency data not shown]. Comprehensive clinical information was collected at each patient visit, including history, physical exam and clinical laboratory values [10]. As shown in Table 4, and consistent with the known demographics of JIA [35], SJIA patients are younger than POLY patients and are gender-balanced, whereas there are more female than male POLY patients. As expected, flare (F) patients from both SJIA and POLY cohorts differ significantly from quiescent (Q) patients for variables reflecting active inflammation: erythrocyte sedimentation rate (ESR), white blood cell count (WBC), platelets (PLT) and joint count (JC, number of affected joints).

**Table 1:** Systemic scoring system for SJIA patients.

Score	Severity level	Systemic symptoms
**0**	None	No active disease

**1**	Mild	Having any one of the following: rash, fevers < 10 days in the past month, ESR 40 to 90, platelets > 450,000

**2**	Moderate	Having at least three of the following: rash, fever > 10 days in the past month, WBC > 20,000, ESR > 90, platelets > 550,000, d-dimers 250 to 500, elevated fibrinogen

**3**	Severe	Having any one of the following symptoms: pneumonitis, percarditis, pleural effusion, Macrophage Activation Syndrome (MAS)

**Table 2:** Arthritis scoring system for SJIA patients.

Score	Severity level	Arthritis
**A**	None	No joint involvement

**B**	Mild	< 5 active joints

**C**	Moderate	5 to 10 active joints

**D**	Severe	> 10 active joints

**Table 3:** Arthritis scoring system for POLY JIA patients*

Score	Severity level	Arthritis
**0**	None	No joint involvement

**1**	Mild	1 to 10 active joints

**2**	Moderate	10 to 20 active joints

**3**	Severe	> 20 active joints

* Arthritis severity is scored differently for SJIA and POLY JIA patients, because the patterns of joint involvement differ between the two groups [33]. The arthritis scoring system is based on frequency analyses of numbers of active joints in early active SJIA [34] and in active POLY JIA [Sandborg C, not shown]. An active joint is defined as a joint with non-bony swelling, or limited range of motion with either tenderness or pain on motion.

**Table 4:** Subjects demographic and clinical characteristics

Characteristics	SJIA Flare	SJIA Quiescence	POLYJIA Flare	POLYJIA Quiescence
N^1^	22	24	17	8

Female/Male^2^	11/11	11/13	14/3	7/1

African- American	1	2	1	1

Asian	3	3	0	0

Caucasian	8	10	11	4

Caucasian Hispanic	10	9	5	3

Median age (yr) at disease onset (range)	5.8 (1.7 to 15.7)	5.8 (1.4 to 15.7)	8.6 (1.2 to 15.1)	8.6 (1.2 to 15.1)

Median age (yr) at sample collection (range)	9.3 (3.5 to 16.6)	11.1 (2.4 to 18.9)	13.4 (5 to 18.7)	14.1 (6.1 to 19.2)

Median WBC (x103/ul) (range)	13.3 (5.3 to 27.4)^3^	6.9 (3.4 to 11.8)	8.8 (4.9 to 15.1)	7.5 (5.6 to 9.8)

Median platelets (x103/ul) (range)	461 (257 to 814)^4^	280 (170 to 400)	384 (211 to 512)^5^	283 (224 to 432)

Median ESR (mm/h) (range)	81 (11 to 121)^6^	5.5 (0 to 18)	31 (7 to 78)^7^	9 (2 to 15)

Median joint count (range)	9 (1 to 28)^8^	0 (0 to 1)	25 (3 to 62)^9^	0 (0)

Median prednisone dose, mg/kd/day (range)	0.01 (0 to 0.53)^10^	0 (0 to 1.1)	0 (0 to 0.1)	0 (0 to 1)

Methotrexate/ total no. samples (%)	8/22 (36%)	7/24 (29%)	10/17 (59%)	6/8 (75%)

Anti-TNF/total no. samples (%)	7/22 (32%)	9/24 (38%)	5/17 (29%)^11^	5/8 (63%)^11^

IL-1RA/total no. samples (%)	1/22 (5%)	2/24 (8%)	0/17	0/8

F, Flare; Q, Quiescent; SJIA F, systemic score of ≥ 1 and/or an arthritis score of ≥ B; POLY F, arthritis score of ≥ 1 (See Tables 1-3)

WBC: white blood count; ESR: erythrocyte sedimentation rate

^1 ^31 unique SJIA patients, and 18 unique PolyJIA patients; 15 SJIA patients and 7 PolyJIA patients gave samples during flare and quiescence.

^2 ^*P *< 0.05 (chi-square test)

Tests performed using Kruskal-Wallis with Dunn's multiple comparison test post test (3 to 9):

^3 ^*P *< 0.0001 compared to SJIA Q and *P *< 0.05 compared to Poly Q

^4 ^*P *< 0.0001 compared to SJIA Q and *P *< 0.01 compared to Poly Q

^5 ^*P *< 0.05 compared to SJIA Q

^6 ^*P *< 0.0001 compared to SJIA Q and *P *< 0.01 compared to Poly Q

^7 ^*P *< 0.01 compared to SJIA Q

^8 ^*P *< 0.0001 compared to SJIA Q and Poly Q

^9 ^*P *< 0.0001 compared to SJIA Q and Poly Q

^10 ^One patient received intravenous methylprednisolone pulse of 1 g daily for three days at the time of the sample.

^11 ^This difference may reflect inclusion of subsets of patients who are anti-TNF responders and non-responders. This difference also raises the possibility that the gene signatures could be specifically influenced by differential response to this drug in these groups.

### Sample processing

Blood samples were obtained only when there was a clinical need for blood tests. A total of 3 to 4 ml of blood was collected directly in vacutainer cell preparation tubes (CPT) with sodium citrate (Becton Dickinson, Franklin Lakes, NJ, USA). Peripheral blood mononuclear cells (PBMCs) were isolated within three hours of collection by centrifugation of CPT tubes, per the manufacturer's instructions.

### RNA preparation

Purified PBMCs were lysed in RLT reagent (Qiagen, Valencia, CA, USA) and lysate was stored at -80°C until RNA extraction. RNA was isolated using the RNeasy mini kit (Qiagen), per the manufacturer's instructions with an additional on-column DNase I (Qiagen) treatment for 40 minutes. The RNA concentration was measured by the Ribogreen assay (Molecular Probes, Grand Island, NY, USA) or by absorbance at 260 nm. The purity of RNA was assessed by the ratio of the absorbance readings at 260 and 280 nm. The integrity of the RNA samples was also checked by either agarose gel electrophoresis or with the Agilent 2100 Bioanalyzer (Agilent Technologies, Santa Clara, CA, USA).

### Gene panel selection

In a pilot study, paired flare/remission PBMC samples from 14 SJIA patients were processed for RNA as described [36] and analyzed using Lymphochip cDNA microarrays (Patrick Brown, Stanford University, Stanford, CA, USA) [37,38]. A large number of genes were identified as differentially expressed in flare versus remission samples by Significance Analysis of Microarrays (SAM) [39]. Hierarchical clustering was performed with the Cluster program and visualized using TreeView [40] (Eisen Lab, University of California, Berkeley, CA), as illustrated for a subset of genes in Additional file 1, and also in [36,41]. The full data set, GSE37388, is released to the public on the Gene Expression Omnibus (GEO) database. From the large set, we selected genes (n = 131) representing various ontologic categories, such as signaling, transcription, inflammation and immune function. We then added other immune-related genes (n = 50) that are expressed in PBMC and implicated in JIA or RA by published reports. The genes were selected prior to analysis of any blood samples for this study, and the samples used for the microarray experiment were not re-used here. The 181 selected genes are shown on Additional file 2; we confirmed that many are immune-related using the program PANTHER 7.0 (Protein ANalysis THrough Evolutionary Relationships) Classification System (Thomas Lab, University of Southern California, Los Angeles, CA, USA), which classifies proteins by their functions, using published experimental evidence and evolutionary relationships [42] (http://www.pantherdb.org/) to categorize their biological functions. This analysis showed that the largest functional category is inflammatory chemokine and cytokine signaling pathways (14.6% of the genes), followed by interleukin signaling pathways (10.8%), apoptosis signaling pathways (9.9%) and toll receptor signaling pathways (6.2%). A full list of categories covered is shown on Additional file 3.

### Gene expression detection by kinetic PCR

The kinetic RT-PCR assay was performed as described [43]. Briefly, all reactions were carried out in duplicate as a single-step RT-PCR reaction, using SYBR green chemistry. Data from duplicate reactions for each gene were averaged and normalized based on levels of expression of four housekeeping genes: eukaryotic translation elongation factor 1 alpha1 (EEF1A1), protein phosphatase 1, catalytic subunit, gamma isoform (PPP1CC), ribosomal protein L12 (RPL12), and ribosomal protein L41 (RPL41). The normalized expression level, housekeeping normalized units, of each gene was used to determine the fold change among samples. In a preliminary experiment, we found that a subset (n = 75) of our gene panel showed very limited variation in level (± 2-fold difference from the mean value) in five healthy individuals (two females and three males) over a four-month period (data not shown).

### Identification of ESR or JC significantly associated genes in SJIA and POLY

Genes significantly associated with SJIA and POLY were determined using Pearson's correlation and Student's *t*-test, as explained in the Results section.

### Significance analysis of the canonical biological pathways

The biological pathways indicated by the group of genes associated with each clinical parameter/patient cohort subset were determined by pathway analysis with Ingenuity IPA system (Ingenuity Systems, Redwood City, CA, USA; http://www.ingenuity.com). The significance of either ESR or JC related pathways was analyzed using sparse linear discriminant analysis method, as previously described [44]. Correlation between SJIA ESR-related and JC-related pathways was analyzed by Pearson correlation. To determine a threshold to extract pathways that significantly differentiate ESR and JC in SJIA, 500 simulated SJIA ESR-related and 500 simulated JC-related pathway data sets were created by permutation of canonical pathway identifications and their associated pathway *P-*values for SJIA ESR or JC. For each canonical pathway, the absolute *P-*value difference in logarithm form between SJIA ESR and JC was computed using one of the 500 simulated SJIA ESR and one of the 500 simulated JC pathway *P*-value data sets. This led to 500 absolute log *P-*value differences for each canonical pathway between SJIA ESR and JC, which later were sorted and 20%, 50% and 80% values were computed. Densities of the absolute differences between SJIA ESR and JC-related pathways for the original and the simulated data sets (20%, 50%, and 80%) were plotted using the R package. Comparison of the original data set and the 80^th ^percentile simulated data set determined the threshold to select significantly different pathways between SJIA ESR and JC. A similar approach was applied to the analysis of significantly different pathways between SJIA ESR and POLY ESR.

## Results

### ESR and JC-associated gene expression in JIA

ESR was chosen as a quantitative measure of systemic inflammation for our analysis, as it typically rises in association with flares of systemic symptoms and was assessed in the largest number of samples. We also considered another measure of systemic inflammation, C-reactive protein (CRP), but few samples were assessed for CRP, precluding the use of this parameter in our analysis. The number of affected counts (joint count, JC), as defined above (Introduction), was used as a quantitative measure of arthritis.

**Figure 1:**
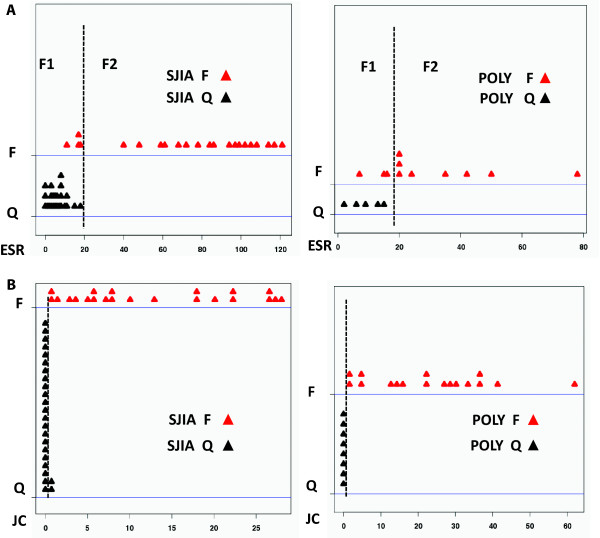
**Distribution of ESR or JC values by Flare (F) or Quiescence (Q) disease states**. **(A) **The SJIA/POLY F patients are partitioned into two groups (F1 and F2) by ESR value < 20. **(B) **SJIA/POLY JC values are generally non-overlapping, in accordance with the F and Q disease states.

Our samples were initially classified as flare or quiescence based on criteria that we have developed for analysis of JIA (Tables 1, 2 and 3), as previously published [10], and ESR and JC are part of these criteria. We performed a distribution analysis of ESR or JC values by disease states (flare/quiescence) using R Epicalc package (http://cran.r-project.org/web/packages/epicalc/) to investigate if additional subgroups would be revealed. Visual inspection of the results show that the SJIA and the POLY flare patients could be partitioned into two groups related to their ESR values (Figure 1A): F1, with ESR values below 20, and F2, with ESR values above 20. All patients in the F1 subgroup had mild flares by our other criteria (not shown). Quiescence samples all had ESR below 20, clustering together with the F1 flare group. This analysis also showed that, in our samples, JC values in the flare and quiescence disease states are generally non-overlapping in both SJIA and POLY patients, with quiescence samples with 0 or 1 joint count, and all flare samples above zero (Figure 1B).

**Figure 2:**
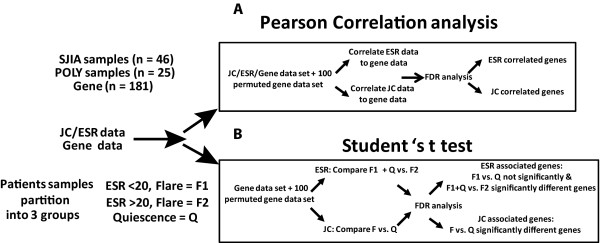
**Schematic of the experimental design and result summary**. **(A) **Pearson correlation analysis and **(B) **Student's *t*-test were used to identify ESR or JC associated genes in SJIA and POLY.

We analyzed the association of the 181 gene panel with ESR and JC in both SJIA and POLY samples, using the strategies delineated in Figure 2. Genes whose expression was significantly associated with ESR or JC in SJIA and POLY cohorts were identified in two ways. As described in Figure 2A, Pearson correlation analyses were performed to correlate ESR or JC values with patient expression data sets. To assess the significance of these findings, we calculated the global false discovery rate (gFDR) by 100-fold permutation of normalized kPCR data. After determining the gFDR, local FDR (lFDR) analysis can compute and assign significance measures to all features [45]. A cut-off value of lFDR ≤ 0.05 was used to select significant genes for downstream pathway analysis.

We also analyzed gene expression association using Student's *t*-test, as shown in Figure 2B. For ESR, based on the analysis from Figure 1A, we initially divided our samples into three groups: flare samples with ESR < 20 (F1), flare samples with ESR > 20 (F2), and quiescence (to ensure that differences between F1 and Quiescence were not overlooked). We identified genes whose mean expression value differed significantly between the F1 (ESR < 20) and F2 (ESR > 20) patient groups, but no differences in genes expressed by the F1 and the quiescence group were found. Subsequently, we grouped the flare F1 and the quiescence groups into one group for ESR analysis. For JC, no other partitioning was necessary, as shown in Figure 1B, and samples were grouped into flare and quiescence groups. As we did previously for Pearson analysis, we calculated local FDR and a value of < 0.05 was considered significant (Figure 2B). This second analysis found genes missed by correlation analysis, as the latter requires a linear relationship and captures genes with more tightly regulated expression (small differences between F and Q samples).

Pearson correlation analysis of expression data from SJIA subjects found 79 genes from our panel to be ESR-correlated and 36 genes to be JC-correlated. Student's *t*-test found 66 ESR-associated and 79 JC-associated genes in SJIA. This pattern differed from relationships of the expression levels of the same genes with these clinical parameters in POLY-course JIA patients: 20 ESR-correlated and no JC-correlated genes were found in POLY, and none of the genes were ESR-associated or JC-associated by Student's *t*-test in POLY. Combining both analyses, we found 91 ESR-related and 92 JC-related genes in SJIA, and 20 ESR-related and no JC-related genes in POLY. A list of significantly associated genes is on Additional file 4. Additional file 5 (Supplementary Figure 2A-F) diagrams the fold changes in expression of the selected genes between groups (for example, F2 versus F1 + Q) and between quartiles of ESR or JC. The probability density analysis graphically represents the normalized frequency distribution of the fold ratio of the selected two groups. This result indicates that our selected genes have significant variation between groups (limited variation = fold ratio close to 1) while showing strong association. This analysis further supports our approach (Figure 2) for the identification of significant associations. The reduced number of genes associated with these clinical parameters in the POLY cohort was not surprising given that the gene list was chosen in large part using expression data from SJIA PBMC. Indeed, this finding implies a degree of specificity of the associated genes for SJIA (see discussion). Another likely contribution to this difference might be the extent that disease-related processes are reflected in peripheral blood in the two disease types.

### Comparative analysis of SJIA ESR and JC related pathways

**Figure 3:**
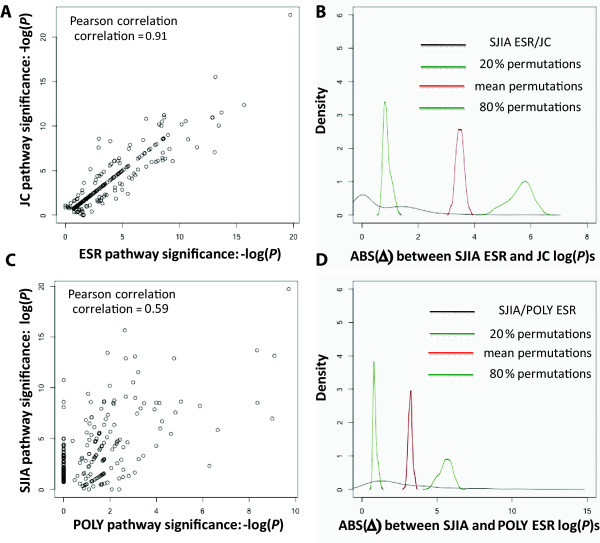
**Analyses of biological pathways associated with SJIA ESR, SJIA JC and POLY ESR**. Comparative analyses determine pathways that are significantly different in their association with SJIA ESR vs JC (top panels) or that differentiate SJIA/ESR and POLY/ESR cohorts (bottom panels). **(A) **Pearson correlation analysis between SJIA ESR and JC related pathways identified by Ingenuity Analysis. **(B) **Simulation analyses to determine the threshold for significantly differentiating pathways between ESR and JC in SJIA. Densities of the absolute differences between SJIA ESR- and JC-related pathways, for the original and the simulation data sets (20%, 50% and 80%) are shown. See Methods for more details. **(C) **Pearson correlation between SJIA and POLY ESR-related pathways identified by Ingenuity Analysis. **(D) **Simulation analysis to determine the threshold for significantly differentiating pathways between SJIA and POLY ESR, performed as in (B).

Using the lists of associated genes, we determined biological pathways associated with each clinical parameter/patient cohort by pathway analysis with the Ingenuity IPA system. ESR-related and JC-related pathways were then compared, to investigate whether the same biological pathways are involved in ESR and JC elevations in SJIA. As shown in Figure 3A, there is strong correlation (Pearson correlation coefficient, 0.91) between SJIA ESR and JC-related pathways (n = 189), implying that some of the same pathways play roles in the systemic and arthritic components of the disease. Shown in Figure 3B, densities of the absolute log *P-*value differences of all pathways between SJIA ESR and JC, for the original and the 20, 50 and 80 percentile of the simulated random data sets, were computed and plotted.

Significantly differentiating pathways between ESR and JC in SJIA revealed by this analysis are in Table 5 (top two pathways), as were pathways that were correlated comparably with both ESR and JC (Table 5). The only pathway more significantly related to SJIA ESR than to SJIA JC was the glucocorticoid (GC) receptor signaling pathway. The expression of most of the genes in this pathway was higher in samples with higher ESR compared to samples with lower ESR. In contrast, the PI3K/Akt signaling pathway was more significantly related to SJIA JC. Though the significance of the association favored JC, the expression levels of most genes in this cell survival pathway were higher in samples with higher ESR or higher JC. However, as might be expected, TP53, which encodes p53, a pro-apoptotic, negative regulator of the Akt pathway [46], was down-regulated in association with JC and ESR elevations. Consistent with these results, we have previously reported that purified monocytes have lower TP53 transcript levels and increased cellular resistance to apoptotic stimuli during SJIA flare compared to quiescence [36]. Overall, the identification of some pathways that are differentially correlated with ESR and JC raises the possibility of differences in aspects of the immunobiology of arthritis compared to systemic inflammation in SJIA, as discussed below.

**Table 5:** Biologic pathways that distinguish or are shared between SJIA ESR and SJIA JC

IPA Canonical Pathways	-Logarithm (*P*-value)		Difference (ESR-JC)	Gene expression with higher ESR/JC*
			
	SJIA ESR	SJIA JC		
Glucocorticoid receptor signaling	5.68	3.07	2.61	Higher (both)

PI3K/AKT signaling	1.27	3.73	-2.45	Higher (both)

Role of PKR in interferon induction and antiviral response	4.43	4.43	0	Higher (both)

Altered T cell and B cell signaling in rheumatoid arthritis	3.91	3.91	0	Higher (both)

T helper cell differentiation	3.74	3.74	0	Lower (both)

iCOS-iCOSL signaling in T helper cells	3.73	3.73	0	Lower (both)

CD40 signaling	3.73	3.73	0	Lower (both)

MIF regulation of innate immunity	3.70	3.70	0	Higher (both)

LPS-stimulated MAPK signaling	3.42	3.42	0	Higher (both)

CD27 signaling in lymphocytes	3.18	3.18	0	Higher (both)

MIF-mediated glucocorticoid regulation	3.02	3.02	0	Higher (both)

Role of cytokines in mediating communication between immune cells	3.01	3.01	0	Higher (both)

TREM1 signaling	2.91	2.91	0	Higher (both)

Crosstalk between dendritic cells and natural killer cells	2.81	2.81	0	Lower (both)

Docosahexaenoic acid (DHA) signaling	2.76	2.76	0	Higher (both)

Regulation of IL-2 expression in activated and anergic T lymphocytes	2.39	2.39	0	Higher (both)

Toll-like receptor signaling	2.37	2.37	0	Lower (both)

fMLP signaling in neutrophils	2.33	2.33	0	Higher (both)

TNFR1 signaling	2.22	2.22	0	Higher (both)

RANK signaling in osteoclasts	2.16	2.16	0	Lower (both)

4-1BB signaling in T lymphocytes	2.12	2.12	0	Higher (both)

CD28 signaling in T helper cells	2.11	2.11	0	Lower (both)

Communication between innate and adaptive immune cells	2.06	2.06	0	Higher (both)

T cell receptor signaling	2.03	2.03	0	Lower (both)

ILK signaling	2.01	2.01	0	Higher (both)

* in ≥ 50% of the genes in the pathway; in comparison to lower ESR/JC.

A number of pathways were significantly related to SJIA ESR and JC to the same degree (Table 5). For several of these pathways, the expression of most of the associated genes was higher in samples with higher ESR or JC. These pathways include, among others, protein kinase receptor (PKR, a pattern-recognition receptor) signaling in interferon induction, T cell and B cell signaling in the pattern of rheumatoid arthritis (RA), and (macrophage) migration inhibition factor (MIF) regulation of innate immunity. Other genes in pathways associated with activating innate responses, such as lipopolysaccharide (LPS) signaling and triggering receptor expressed on myeloid cells (TREM1) signaling are also higher samples with either higher ESR or JC. Genes in other pathways showed lower expression in samples with higher ESR or JC, such as T helper cell differentiation, iCOS-iCOSL (inducible T-cell co-stimulator/ligand) signaling in T helper cells and CD40 (co-stimulatory molecule on antigen presenting cells) signaling. Notably, these down-regulated pathways are associated with adaptive immune responses. Also down-regulated in association with elevations of both SJIA ESR and JC is the pathway for crosstalk between dendritic cells and natural killer cells, which can be involved in restriction of innate responses [47].

Two genes, the DNA repair enzyme ATM and the transcription factor NFATC2 (also known as NFAT1), are in the pathway for RANK signaling in osteoclasts and are both down-regulated in association with systemic (ESR) and arthritic (JC) disease activity. The Rank/RankL pathway is an important regulator of bone remodeling [48]. An ATM deficiency has been described in CD4+ T cells from rheumatoid arthritis (RA) patients [49], associated with premature immunosenescence. However, ATM may also be involved in bone formation, and ATM deficient animals show increased numbers of osteoclasts [50]. The transcription factor NFATC2 has been identified as a negative regulator of cartilage cell growth [51]. It is also important in T cell effector function, translocating to the nucleus following T cell receptor activation and regulating expression several cytokines in CD4 T cells (reviewed in [52]). Thus, its inverse correlation with ESR and JC may be similar to the other T cell-related pathways described above. Interestingly, hyperactivation of NFATC2 in T cells is associated with decreased susceptibility to experimental autoimmune encephalomyelitis, indicating that increased NFATC2 activity may have immunomodulatory effects that down-regulate autoaggressive reactions [53].

### Comparative analysis of SJIA and POLY ESR-related pathways

We next asked whether some biological pathways involved in SJIA ESR elevation are also involved in POLY ESR elevation, by comparing SJIA and POLY ESR-related genes. As shown in Figure 3C, there is reduced correlation (correlation coefficient, 0.59) between SJIA and POLY ESR-related pathways (n = 119), compared to the correlation we observed between SJIA ESR- and SJIA JC-related pathways. Shown in Figure 3D, densities of the absolute logarithm *P-*value differences of all pathways between SJIA and POLY ESR, for the original and the 20, 50 and 80 percentile of the simulated random data sets, were computed and plotted.

Several pathways differ significantly between SJIA and POLY ESR, as quantified by absolute difference between the SJIA and POLY pathways (Table 6). These include: the role of macrophages, fibroblasts and endothelial cells in RA, IL-10 signaling, glucocorticoid receptor signaling, among others. These data suggest a greater role for these pathways in SJIA, compared to POLY. However, very few genes were associated with POLY ESR in most pathways, resulting in low significance of association with POLY (not shown). In these differentiating pathways, the (few) genes correlating with ESR were higher in POLY samples with higher ESR values, suggesting that these genes, perhaps in the context of other pathways or in the context of the identified pathways but within the joint, contribute to inflammation in polyarticular course JIA. Indeed, evidence from RA, the adult disease most similar to polyarticular JIA, implicates monocyte and macrophage activation [54] and endothelial cell dysfunction [55], both in joints and in the periphery.

**Table 6:** Biologic pathways that distinguish or are shared between SJIA ESR and POLY ESR

IPA Canonical Pathways	-Logarithm (*P*-value)		Difference (SJIA-POLY)	Gene expression with higher SJIA ESR/ POLY ESR
			
	SJIA ESR	POLY ESR		
IL-10 signaling	8.56	4.22	4.34	Higher (both)

Role of macrophages, fibroblasts and endothelial cells in rheumatoid arthritis	6.80	1.14	5.65	Higher (both)

Role of osteoblasts, osteoclasts and chondrocytes in rheumatoid arthritis	5.84	0.82	5.01	Higher (both)

Glucocorticoid receptor signaling	5.68	1.34	4.34	Higher (both)

B cell receptor signaling	5.60	2.07	3.53	Higher (both)

Role of PKR in interferon induction and antiviral response	4.43	0.86	3.56	Higher (both)

Systemic lupus Erythematosus signaling	4.08	0.46	3.62	Higher (both)

altered T cell and B cell signaling in rheumatoid arthritis	3.91	1.34	2.57	Higher (both)

A proliferation-inducing ligand mediated signaling	3.80	0.95	2.86	Lower (both)

IL-15 signaling	3.76	0.76	3.01	Lower (both)

T helper cell differentiation	3.74	0.00	3.74	Lower (both)

iCOS-iCOSL signaling in T helper cells	3.73	0.51	3.22	Lower (both)

CD40 signaling	3.73	0.75	2.98	Lower (both)

B cell activating factor signaling	3.70	0.93	2.77	Lower (both)

Dendritic cell maturation	3.65	0.94	2.71	Higher (both)

IL-12 signaling and production in macrophages	3.08	0.52	2.55	Lower (both)

p38 MAPK signaling	3.08	0.52	2.55	Higher (both)

PPAR alpha/RXR alpha activation	2.96	0.42	2.55	Higher (both)

* in ≥ 50% of the genes in the pathway; in comparison to lower SJIA ESR/POLY ESR.

As observed in the previous analysis, pathways associated with T cell responses are significantly associated with ESR in SJIA but the genes in these pathways show lower expression in samples with higher ESR in comparison to samples with lower ESR. In addition, this analysis showed that genes in B cell activating factor (BAFF) signaling, April (A proliferation-inducing ligand, TNFSF13)-mediated signaling and IL-15 signaling pathways show lower expression samples with in elevated ESR in SJIA (Table 6).

### Comparative analysis of joint count (JC) correlated genes in systemic and arthritis phase (SAF) and arthritis phase (AF) SJIA patients

**Figure 4:**
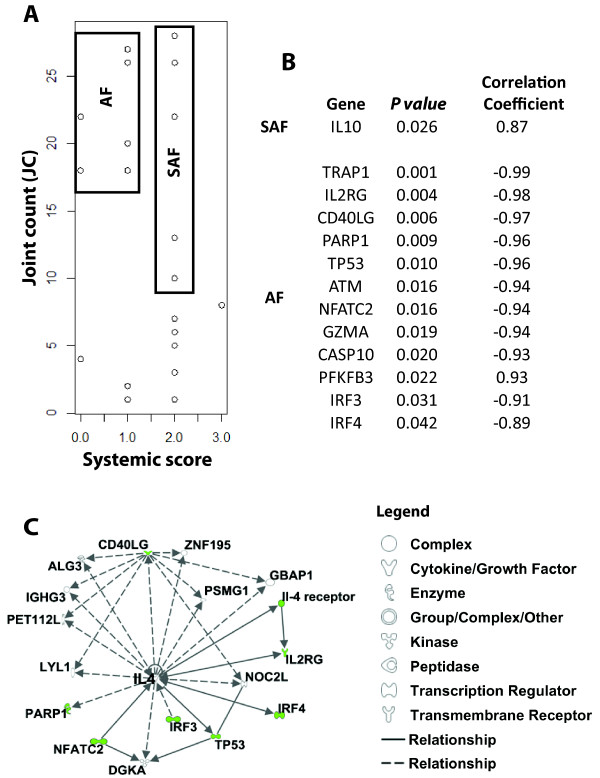
**Analysis of JC-associated genes in SJIA patients with high JC counts but different systemic scores**. **(A) **Distribution analysis of SJIA patients, according to both joint count (JC) and systemic scores, established systemic plus arthritis flare (SAF) and arthritis flare (AF) subgroups. **(B) **Pearson correlation analysis relating JC values to the associated gene expression in both the SAF and AF subgroups. **(C) **Pathway analysis of the JC correlated genes in AF subgroup. Differentially JC-correlated genes were analyzed using data mining software (Ingenuity Systems) to identify relevant biological pathways associated with each clinical subgroup. The node color indicates the degree of positive (red) or negative (green) correlation to SJIA AF. Nodes are displayed using shapes that represent the functional class of the gene product and different relationships are represented by line type (see legend). Relationships are primarily due to co-expression, but can also include phosphorylation/dephosphorylation, proteolysis, activation/deactivation, transcription, binding, inhibition, biochemical modification.

Using the previously identified JC-associated genes (Additional file 4), we then investigated whether arthritis-related gene pathways change when the disease phenotype changes from the earlier systemic and arthritic activity/flare (SAF) phase to arthritis-only activity/flare (AF) phase. Shown in Figure 4A, SJIA patients were distributed according to values of JC and systemic scores (Tables 1 and 2-) to identify SAF and AF subgroups. Figure 4B shows the JC associated genes that are significantly correlated with JC in SAF and AF subgroups. Within the SJIA SAF group, only IL-10 was identified to positively correlate with JC (*P-*value 0.026). In contrast, 12 genes were found to significantly correlate (negatively) with JC in AF subgroup (Figure 4B, listed in order of decreasing significance): TRAP1, IL2RG, CD40LG, PARP1, TP53, ATM, NFATC2, GZMA, CASP10, PFKFB3, IRF3 and IRF4. Canonical pathway analysis (Figure 4C) mapped 8 of the 12 JC-correlated genes to a single network with the Th2 cytokine, IL-4, at its center. These functional relationships suggest that lack of IL-4 may contribute to arthritis in the AF subgroup. The difference in JC-correlated genes/pathways between AF and SAF supports the hypothesis that different biological pathways are engaged in the chronic arthritis stage versus the more acute (or systemic symptom-associated) arthritis of SJIA.

## Discussion

In this study, we sought to identify molecular pathways involved in the systemic and arthritic components of SJIA by investigating the gene expression pathways associated with increases in ESR and active joint count. We chose ESR as a marker of systemic inflammation, but we note that SJIA flares associated with elevated ESR may also include arthritis. Further, SJIA flares with macrophage activation syndrome (MAS) may actually lower ESR from fibrinogen consumption as part of the coagulopathy [56]. The latter issue does not confound our analysis, as the three flare samples with low ESR were from patients with mild flares without MAS. Strictly speaking, our approach delineated gene associations with ESR; however, in our group of SJIA samples, ESR typically correlated closely with other evidence of systemic disease.

Several variables that influence transcriptional profiles should be considered in relation to our results. It is possible that some of the observed differences in gene expression are due to differences in cell type composition of PBMC between SJIA and POLY, or between flare and quiescence [10]. Changes in abundance of cell types may be relevant to disease mechanisms. For monocyte-related genes, we [41] and others [9] have shown that the differences in transcript abundance are not explained by differences in monocyte numbers alone, but reflect activation state. The use of medication and disease duration at the time of sampling may influence the pattern of gene expression. A larger, likely multi-center, study will be needed to rigorously control for these important variables.

Our analysis revealed overlap in molecular pathways involved in increased ESR and elevated JC in SJIA. This result was not unexpected, given reported correlations between these two parameters [30,57]. However, the glucocorticoid receptor (GCR) signaling pathway was more significantly related to ESR than JC. Systemic symptoms of SJIA respond to exogenous steroids, suggesting the elevation of GCR signaling may represent an endogenous effort to dampen systemic inflammation. The comparable doses of exogenous steroids in the F and Q groups make it less likely that steroid therapy is inducing this pathway. Notably, polymorphism in the GCR gene is associated with the level of inflammatory activity in JIA [58]. Involvement of GCR signaling in systemic inflammation in SJIA and stronger association of this pathway with inflammation in SJIA versus POLY (at least as reflected in blood cells) is consistent with reduced responses in SJIA patients to non-glucocorticoid drugs that are efficacious in subsets of POLY patients (for example, methotrexate and anti-TNFα [59,60]).

We also found that the PI3K/Akt signaling pathway is more significantly related to SJIA JC than ESR. This pathway, which is activated by a variety of stimuli, including IL-1β, TNFα and IL-6, is potentially involved in IL-17 production [61]. IL-17 could be an important factor in SJIA arthritis [19], particularly in the later phase. We did not assess expression of IL-17 in this study, but our preliminary data suggest that CD4+ T cells from SJIA patients secrete higher levels of IL-17 than control cells when cultured in TH17-polarizing conditions [Wong M, Mellins E, unpublished results]. Recently, enrichment of Th17 (and Th1) cells in blood of SJIA patients has been described [62].

Our findings are consistent with the hypothesis that dysregulation of the innate immune system makes a more prominent contribution to SJIA immunopathology than alterations of the adaptive immune system [17,63], whereas adaptive responses are thought to drive oligoarticular and polyarticular JIA [64,65]. However, our results implicate deficiencies in genes associated with T cell-related responses in SJIA pathology, similar to observations in other studies [29]. For example, reduced cytolytic cell activity [66] and diminished function of T regulatory cells may play roles in SJIA etiology [67]. Down-regulated genes associated with cytolytic function also participate in dendritic cell/NK cell and monocyte/NK cell interaction. Some cytolysis genes are part of the IL-15 signaling pathway, and IL-15 is involved in the development of NK cells [68].

In the systemic plus arthritic stage of SJIA, we found that expression of IL-10 in PBMC was positively associated with arthritis. In *in vitro *studies of SJIA monocytes, we and others [26,29] observe that IL-10 is expressed after TLR stimulation, and IL-10 signaling is intact [Macaubas *et al*., unpublished]. Given the immunosuppressive effect of IL-10, association of this gene with arthritis in SJIA may represent an attempt by the immune system to reduce inflammation. The level of IL-10 may be inadequate to deal with the inflammatory challenge, as the frequency of a promoter allele associated with low IL-10 expression is increased in SJIA patients [69,70]. We found that LPS-induced production of IL-10 protein in SJIA monocytes is comparable to controls [41].

A striking finding of this study is that deficiency in IL-4-related pathways correlates with JC in the arthritic phase of SJIA. IL-4 has been implicated in protection against arthritis. Polymorphism in the IL4Rα gene that confers reduced responsiveness to IL-4 is associated with worse outcome in RA [71]. Low levels of circulating IL-4 are observed in patients with active POLY [72]. IL-4 has been shown to suppress growth factor-induced proliferation of cultured rheumatoid synovial cells by interfering with the cell cycle and by decreasing cell survival [73]. In the murine model of collagen-induced arthritis, IL-4 is protective against cartilage and bone destruction [74], and neutralization of IL-4 in the same model results in reversal of arthritis suppression [75]. IL-4 is also protective in the model of proteoglycan-induced arthritis [76]. Interestingly, in proteoglycan-induced arthritis, mice deficient in IL-4Rα showed higher IL-1β, IL-6 and MIP1a, whereas levels of IFNγ and autoantibodies were less affected. These results imply that IL-4 suppresses innate immune activity more than the adaptive system in this arthritis model [77]. This might model the arthritis of late stage SJIA.

IL-4 inhibits expression of pro-inflammatory cytokines, such as IL-1β, TNFα [78,79] and IL-17 [80]. As mentioned, IL-17 is an attractive candidate for a driver of inflammation in the later arthritic phase of SJIA. Th17 cells may become IL-1 independent in SJIA, as seen in an animal model [81]. The IL-1β independence of IL-17 action would be consistent with the decreased efficiency of anti-IL1 therapy in the later arthritic phase of SJIA [19]. The ability of IL-4 to suppress reactivation of committed Th17 cells [82] may be another mechanism by with IL-4 deficiency could contribute to arthritis in SJIA. Finally, in a small, open label study, oral histone deacetylase inhibitors in patients with mean SJIA duration of five years showed significant therapeutic benefit, specifically for arthritis [83]. This finding is consistent with the idea that distinct biology may be involved in later phase arthritis in SJIA.

We found no gene association or correlation linked with POLY joint count, and a limited number of somewhat different genes were associated with elevated ESR in POLY-JIA subjects. Our gene panel was largely derived from a SJIA-based microarray study and, as such, it has a significant bias towards SJIA-related genes. Further, the systemic nature of SJIA predicts more changes in peripheral blood than for POLY, where pathology is more localized. Our POLY cohort was itself heterogeneous, including RF+ and RF- patients, which were analyzed as one group. Most gene expression studies have analyzed RF- patients only [29,84,85]; some have not determined the RF status [86]. Griffin *et al*. 2009 showed that RF+ and RF- patients can share a similar gene signature [21].

It will be of interest to determine the cell type within PBMC that is responsible for particular transcripts. Based on correlated expression patterns with more lineage specific genes, it is most likely that IL-4 transcripts derive from CD4 T cells; the IL-4 message expression is correlated with expression of CD40LG and IL2RG (not shown). In contrast, the IL-10 expression correlates with expression of IL-1, IL-1-related genes and IL-6 (not shown), suggesting IL-10 transcripts are expressed in monocytes. Further studies are also needed to determine the specificity of the SJIA gene signature in relation to other acute inflammatory diseases, such as bacterial and viral infections and other rheumatologic pediatric diseases [27]. Nonetheless, our current results add to the growing evidence that different molecular mechanisms distinguish SJIA from other JIA subtypes [10,11,26,29,32].

## Conclusions

This study demonstrates that analysis of individual clinical parameters in a complex disease like SJIA may reveal unique and informative molecular associations. In addition to elucidating disease immunopathology, this approach may help identify therapeutic targets and strategies tailored to the different phases of SJIA.

## Abbreviations

AF: arthritis-predominant phase; AOSD: Adult Onset Still Disease; BAFF: B cell activating factor; CPT: cell preparation tubes; CRP: C-reactive protein; EEF1A1: eukaryotic translation elongation factor 1 alpha1; ESR: erythrocyte sedimentation rate; F: SJIA flare; FDR: false discovery rate; GEO: Gene Expression Omnibus; GC: glucocorticoid; GCR: glucocorticoid receptor; IL: interleukin; JC: joint count; LPS: lipopolysaccharide; MAS: macrophage activation syndrome; PANTHER: Protein ANalysis THrough Evolutionary Relationships) Classification System; PBMC: peripheral blood mononuclear cells; PCR: polymerase chain reaction; PKR: protein kinase receptor; PLT: platelets; POLY: polyarticular juvenile idiopathic arthritis; PPP1CC: protein phosphatase 1, catalytic subunit, gamma isoform; Q, SJIA quiescence; qPCR: quantitative PCR; RA: rheumatoid arthritis; RF: rheumatoid factor; RPL12: ribosomal protein L12; RPL41: ribosomal protein L41; SAF: systemic plus arthritic phase; SAM: Significance Analysis of Microarrays; SJIA: systemic onset juvenile idiopathic arthritis; WBC: white blood count.

## Competing interests

The authors declare that they have no competing interests.

## Authors' contributions

XBL performed the statistical analysis and wrote the paper. CM performed statistical analysis, interpreted the data and wrote the paper. HCA, S-YPC and ABB designed and performed the kinetic PCR. QW and EC performed statistical analysis. YS and CD processed patients' samples, prepared RNA, performed and analyzed the microarrays. K-HP, RL, C-JL and SHP performed microarray and initial data analysis. TL and CS provided patients' samples and clinical information. SNC helped design the study and the initial strategy for data analysis. EM contributed to study design, interpreted data and wrote the manuscript. All authors read and approved the final manuscript.

## Pre-publication history

The pre-publication history for this paper can be accessed here:

http://www.biomedcentral.com/1741-7015/10/125/prepub

## Supplementary Material

Additional file 1 **Supplementary Figure 1**. Unsupervised hierarchical clustering analysis of a subset of differentially expressed genes from SJIA flare and quiescence samples studied by microarray. Paired samples from 14 subjects at flare (red) and quiescence (green) were studied. Independent samples from the same individual are indicated by *. Each column represents a separate sample; each row represents a separate gene. Click here for file

Additional file 2 **Supplementary Table 1**. The 181 immune-related genes analyzed in this study. Click here for file

Additional file 3 **Supplementary Table 2**. Annotation of the 181 analyzed genes into different functional categories using PANTHER software. Click here for file

Additional file 4 **Supplementary Table 3**. SJIA ESR, SJIA JC and POLY ESR related gene lists. Click here for file

Additional file 5 **Supplementary Figure 2**. The fold changes in expression of the genes that differ between groups and between quartiles of ESR or JC. **(A) **The probability density of fold changes with the selected ESR-related genes between F1 + Q and F2 of SJIA; **(B) **The probability density of fold changes with the selected ESR-related genes between quartiles (the first quartile to the third quartile) of SJIA; **(C) **The probability density of fold changes with the selected JC-related genes between Q and F of SJIA; **(D) **The probability density of fold changes with the selected JC- related genes between quartiles (the first quartile to the third quartile) of SJIA; **(E) **The probability density of fold changes with the selected ESR-related genes between F2 and F1 + Q of POLY; **(F) **The probability density of fold changes with the selected ESR-related genes between quartiles (the first quartile to the third quartile) of POLY. Click here for file
