# Supplementary material for: Correlation analyses of clinical and molecular findings identify candidate biological pathways in systemic juvenile idiopathic arthritis
Source: BMC Med. 2012 Oct 23;10:125. doi: 10.1186/1741-7015-10-125 (PMC3523070; doi:10.1186/1741-7015-10-125)

**Supplementary Figure 1** Unsupervised hierarchical clustering analysis of a subset of differentially expressed genes from SJIA flare and quiescence samples studied by microarray. Paired samples from 14 subjects at flare (red) and quiescence (green) were studied. Independent samples from the same individual are indicated by *. Each column represents a separate sample; each row represents a separate gene.


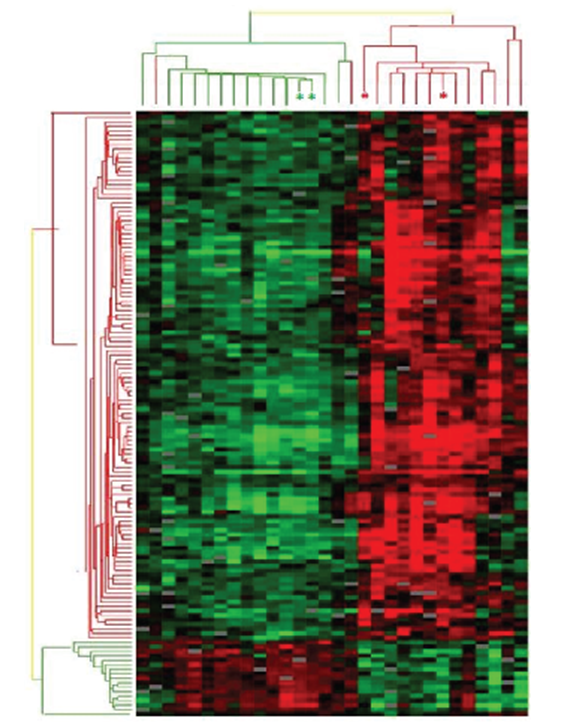

Supplement: Additional file 1 — Supplementary Figure 1. Unsupervised hierarchical clustering analysis of a subset of differentially expressed genes from SJIA flare and quiescence samples studied by microarray. Paired samples from 14 subjects at flare (red) and quiescence (green) were studied. Independent samples from the same individual are indicated by *. Each column represents a separate sample; each row represents a separate gene. [file 1741-7015-10-125-S1.DOC]
