# Supplementary material for: Correlation analyses of clinical and molecular findings identify candidate biological pathways in systemic juvenile idiopathic arthritis
Source: BMC Med. 2012 Oct 23;10:125. doi: 10.1186/1741-7015-10-125 (PMC3523070; doi:10.1186/1741-7015-10-125)
Supplement: Additional file 2 — Supplementary Table 1. The 181 immune-related genes analyzed in this study. [file 1741-7015-10-125-S2.DOC]

**Supplementary Table 1 The 181 immune-related genes analyzed in this study.**

| ABCA1 | CASP10 | FCGR1A | IL12A | IL7 | MXD1 |  | TGFB1 |
| --- | --- | --- | --- | --- | --- | --- | --- |
| ACSL1 | CCL2 | FCGR3A | IL12B | IL8 |  | SELENBP1 | THBD |
| ADM | CCL20 | FNDC3B | IL12RB1 | IL8RB | NFATC2 | SELL | TIA1 |
| ADORA2A | CCR1 | FOSB | IL12RB2 | IRF1 | NFATC3 | SESN3 | TLE3 |
| ADRA2A | CCR4 | FY | IL13 | IRF2 | NFKB2 | SGK | TNF |
| ALDOA | CCR5 |  | IL15 | IRF3 | NFKBIA | SIAH2 | TNFAIP6 |
| ALOX5AP | CD14 | GABARAPL1 | IL16 | IRF4 | NR3C1 | SIRPB | TNFRSF1A |
| ALPL | CD1B | GALC | IL17R | IRF7 |  | SIVA | TNFRSF1B |
| ANPEP | CD40LG | GMPR | IL18 | ISGF3G | PARP1 | SLC11A1 | TNFSF6 |
| ANXA3 | CDC42 | GNLY | IL18BP |  | PBEF1 | SLC2A3 | TP53 |
| AP3S2 | CHI3L1 | GPX4 | IL18R1 | KLF1 | PFKFB3 | SLC7A5 | TRADD |
| AQP3 | CHPT1 | GZMA | IL18RAP |  | PIAS4 | SLPI | TRAF2 |
| ATF3 | CHS1 | GZMB | IL1A | LGALS3BP | PIM1 | SNCA | TRAP1 |
| ATM | CR2 |  | IL1B | LIMK1 | PLAU | SOCS3 | TREM1 |
| AXIN1 | CSF2 | HAVCR2 | IL1R2 | LTA | PLAUR | SP3 | TXN |
| AXUD1 | CTSL | HIF1A | IL1RAP | LTF | POLR2B | SPTB | TYROBP |
|  | CXCL1 | HP | IL1RN |  | PRF1 | STAT1 |  |
| BCL2A1 | CXCL16 |  | IL2 | MAFB | PTGS2 | STAT3 | UBB |
| BCL2L1 |  | ICAM1 | IL2RA | MAP2K3 | PTPNS1 | STAT4 | UBE2J1 |
| BCL3 | EGR1 | ICAM3 | IL2RB | MAPK9 | PTPRJ | STAT5A |  |
| BCL6 | ELAVL1 | ICSBP1 | IL2RG | MCL1 |  | STAT5B | VEGF |
| BNIP3L | ENO1 | IFNB1 | IL4 | MIF | REL | STAT6 | VLDLR |
| BRCA1 |  | IFNG | IL6 | MME | RELA |  |  |
|  | FANCA | IKBKG | IL6R | MMP9 | RELB | TAL1 | ZFP36 |
| CAD | FASLG | IL10 | IL6ST | MPP1 | RNASE2 | TBX21 |  |
