# Supplementary material for: Correlation analyses of clinical and molecular findings identify candidate biological pathways in systemic juvenile idiopathic arthritis
Source: BMC Med. 2012 Oct 23;10:125. doi: 10.1186/1741-7015-10-125 (PMC3523070; doi:10.1186/1741-7015-10-125)
Supplement: Additional file 3 — Supplementary Table 2. Annotation of the 181 analyzed genes into different functional categories using PANTHER software. [file 1741-7015-10-125-S3.DOC]

**Supplementary Table 2 Annotation of the 181 analyzed genes into different functional categories using PANTHER software.**

Categories %

Inflammation mediated by chemokine and cytokine signaling pathway (P00031) 14.6%

Interleukin signaling pathway (P00036) 10.8%

Apoptosis signaling pathway (P00006) 9.9%

Toll receptor signaling pathway (P00054) 6.2%

EGF receptor signaling pathway (P00018) 4.1%

T cell activation (P00053) 3.5%

Wnt signaling pathway (P00057) 3.4%

JAK/STAT signaling pathway (P00038) 3.3%

PDGF signaling pathway (P00047) 3.3%

Ras pathway (P04393) 2.7%

Angiogenesis (P00005) 2.6%

Interferon-gamma signaling pathway (P00035) 2.6%

B cell activation (P00010) 2.6%

Huntington disease (P00029) 2.1%

FAS signaling pathway (P00020) 2.1%

Axon guidance mediated by netrin (P00009) 1.8%

Integrin signaling pathway (P00034) 1.8%

Cytoskeletal regulation by Gho GTPase (P00016) 1.7%

Oxidative stress response (P00046) 1.7%

Parkinson disease (P00049) 1.6%

p53 pathway (P00059) 1.4%

TGF-beta signaling pathway (P00052) 1.4%

Plasminogen activating cascade (P00050) 1.4%

Blood coagulation (P00011) 1.4%

p53 pathway feedback loops 2 (P04398) 1.3%

Hypoxia response via HIF activation (P00030) 1.1%

Axon guidance mediated by Slit/Robo (P00008) 1.0%

Heterotrimeric G-protein signaling pathway-Gi alpha and Gs alpha mediated pathway (P00026) 1.0%

Glycolysis (P000240 1.0%

FGF signaling pathway (P00021) 0.9%

Alzheimer disease-amyloid secretase pathway (P00003) 0.6%

Heterotrimeric G-protein signaling pathway-Gq alpha and Go alpha mediated pathway (P00027) 0.6%

p53 pathway by glucose deprivation (P04397) 0.6%

VEGF signaling pathway (P00021) 0.6%

p53 pathway feedback loops 2 (P04392) 0.6%

Endothelin signaling pathway (P00021) 0.6%

Alzheimer disease presenilin pathway (P00004) 0.4%

Alpha adrenergic receptor signaling pathway (P00002) 0.4%

Fructose galactose metabolism (P02744) 0.4%

Insulin/IGF pathway-mitogen activated protein kinase kinase/MAP kinase cascade (P00889) 0.3%

Pentose phosphate pathway (P02762) 0.3%

Arginine biosynthesis (P02728) 0.1%

Vitamin D metabolism and pathway (P04396) 0.1%

De novo pyrmidine ribonucleotides biosynthesis (P02740) 0.1%
