# Supplementary material for: Correlation analyses of clinical and molecular findings identify candidate biological pathways in systemic juvenile idiopathic arthritis
Source: BMC Med. 2012 Oct 23;10:125. doi: 10.1186/1741-7015-10-125 (PMC3523070; doi:10.1186/1741-7015-10-125)
Supplement: Additional file 4 — Supplementary Table 3. SJIA ESR, SJIA JC and POLY ESR related gene lists. [file 1741-7015-10-125-S4.DOC]

**Supplementary Table 3 SJIA ESR, SJIA JC and POLY ESR related gene lists.**

___________________________________________________________________________________________

SJIA ESR SJIA JC POLY ESR

___________________________________________________________________________________________

Genes combined lFDR* Genes combined lFDR* Genes combined lFDR*

___________________________________________________________________________________________

HP 1.06E-05 SLC7A5 1.80E-05 BCL2A1 6.85E-04

BCL2A1 2.62E-05 GABARAPL1 1.63E-04 EGR1 9.94E-04

ANPEP 4.50E-05 TLE3 1.63E-04 SOCS3 9.94E-04

PIM1 4.50E-05 CXCL16 1.01E-03 TNFAIP6 9.94E-04

BCL3 1.22E-04 THBD 1.01E-03 PTGS2 1.66E-03

CCR1 1.22E-04 ANPEP 6.49E-03 PBEF1 3.62E-03

CDC42 1.22E-04 BCL2A1 6.49E-03 SLC2A3 3.62E-03

IL1RAP 1.22E-04 CDC42 6.49E-03 MAFB 1.72E-02

TREM1 1.22E-04 CXCL1 6.49E-03 MXD1 1.72E-02

UBE2J1 1.22E-04 IL15 6.49E-03 CXCL1 2.27E-02

BCL2L1 4.18E-04 PIM1 6.49E-03 THBD 2.87E-02

BCL6 4.18E-04 SOCS3 6.49E-03 ANPEP 4.44E-02

CXCL1 4.18E-04 BCL3 7.78E-03 CD14 4.44E-02

FCGR1A 4.18E-04 CCR1 7.78E-03 CHPT1 4.44E-02

RNASE2 4.18E-04 HP 7.78E-03 IL1RN 4.44E-02

SLC2A3 4.18E-04 RELB 8.06E-03 RELA 4.44E-02

SOCS3 4.18E-04 SESN3 8.06E-03 RNASE2 4.44E-02

ACSL1 9.99E-04 TP53 8.06E-03 SGK 4.44E-02

ADM 9.99E-04 TRAP1 8.06E-03 TREM1 4.44E-02

IL10 9.99E-04 TGFB1 8.63E-03 ZFP36 4.44E-02

IL1RN 9.99E-04 TREM1 8.63E-03

PBEF1 9.99E-04 BCL6 1.36E-02

SGK 9.99E-04 GNLY 1.36E-02

SNCA 9.99E-04 IL1RAP 1.36E-02

TXN 9.99E-04 IL1RN 1.36E-02

MAFB 1.74E-03 RNASE2 1.36E-02

FNDC3B 1.99E-03 TBX21 1.36E-02

THBD 1.99E-03 UBE2J1 1.36E-02

TNFAIP6 1.99E-03 CD40LG 1.51E-02

CD40LG 2.25E-03 FCGR1A 1.51E-02

FOSB 3.81E-03 IL10 1.51E-02

GALC 3.81E-03 IL2RB 1.51E-02

MCL1 3.81E-03 IL7 1.51E-02

SLC11A1 3.81E-03 PARP1 1.51E-02

ALDOA 4.98E-03 SLC2A3 1.51E-02

ALOX5AP 4.98E-03 ABCA1 1.59E-02

ANXA3 4.98E-03 CASP10 1.59E-02

CD14 4.98E-03 IRF4 1.59E-02

IL8RB 4.98E-03 REL 1.59E-0

MME 4.98E-03 ACSL1 2.04E-02

MMP9 4.98E-03 ADM 2.04E-02

MPP1 4.98E-03 ALDOA 2.04E-02

NFKBIA 4.98E-03 AXUD1 2.04E-02

PFKFB3 4.98E-03 BCL2L1 2.04E-02

PLAUR 4.98E-03 FOSB 2.04E-02

SLPI 4.98E-03 GALC 2.04E-02

TLE3 4.98E-03 GZMA 2.04E-02

VEGF 4.98E-03 IL12RB2 2.04E-02

BNIP3L 5.35E-03 IL18RAP 2.04E-02

PARP1 6.59E-03 IL1B 2.04E-02

GZMA 7.01E-03 IL2RG 2.04E-02

IL16 7.01E-03 IL8RB 2.04E-02

NFATC2 7.01E-03 MCL1 2.04E-02

PTPNS1 7.01E-03 NFKBIA 2.04E-02

TRAF2 7.01E-03 PBEF1 2.04E-02

AXUD1 7.64E-03 PTGS2 2.04E-02

IL2RG 9.46E-03 SGK 2.04E-02

GMPR 9.85E-03 TNFAIP6 2.04E-02

IL1B 9.85E-03 ANXA3 2.25E-02

IL2RB 9.85E-03 MAFB 2.25E-02

KLF1 9.85E-03 MMP9 2.25E-02

MXD1 9.85E-03 NFATC2 2.25E-02

SESN3 9.85E-03 SLPI 2.25E-02

UBB 9.85E-03 SNCA 2.25E-02

ABCA1 1.48E-02 TXN 2.25E-02

ALPL 1.48E-02 ATM 2.37E-02

CHI3L1 1.48E-02 BNIP3L 2.37E-02

HIF1A 1.48E-02 GMPR 2.37E-02

MAP2K3 1.48E-02 IRF3 2.37E-02

TAL1 1.48E-02 KLF1 2.37E-02

PTGS2 1.58E-02 MAP2K3 2.37E-02

CXCL16 1.59E-02 MPP1 2.37E-02

ATM 1.70E-02 SELENBP1 2.37E-02

PRF1 1.70E-02 SIAH2 2.37E-02

STAT4 1.70E-02 SPTB 2.37E-02

TGFB1 1.70E-02 CTSL 3.03E-02

TP53 1.70E-02 UBB 3.03E-02

ATF3 2.20E-02 AXIN1 3.11E-02

NFKB2 2.20E-02 CHI3L1 3.11E-02

SIAH2 2.20E-02 FNDC3B 3.11E-02

SLC7A5 2.20E-02 PFKFB3 3.11E-02

ZFP36 2.20E-02 PRF1 3.11E-02

IL6 2.78E-02 PTPNS1 3.11E-02

AXIN1 2.93E-02 MME 3.27E-02

EGR1 2.93E-02 CHPT1 3.87E-02

ENO1 2.93E-02 ADRA2A 4.45E-02

FASLG 2.93E-02 ALOX5AP 4.45E-02

GABARAPL1 2.93E-02 CCR5 4.45E-02

NFATC3 2.93E-02 HIF1A 4.45E-02

TRAP1 2.93E-02 ICSBP1 4.45E-02

SELENBP1 4.80E-02 PLAUR 4.45E-02

TAL1 4.45E-02

* lFDR: Local False Discovery Rate
