# Supplementary material for: Correlation analyses of clinical and molecular findings identify candidate biological pathways in systemic juvenile idiopathic arthritis
Source: BMC Med. 2012 Oct 23;10:125. doi: 10.1186/1741-7015-10-125 (PMC3523070; doi:10.1186/1741-7015-10-125)
Supplement: Additional file 5 — Supplementary Figure 2. The fold changes in expression of the genes that differ between groups and between quartiles of ESR or JC. (A) The probability density of fold changes with the selected ESR-related genes between F1 + Q and F2 of SJIA; (B) The probability density of fold changes with the selected ESR-related genes between quartiles (the first quartile to the third quartile) of SJIA; (C) The probability density of fold changes with the selected JC-related genes between Q and F of SJIA; (D) The probability density of fold changes with the selected JC- related genes between quartiles (the first quartile to the third quartile) of SJIA; (E) The probability density of fold changes with the selected ESR-related genes between F2 and F1 + Q of POLY; (F) The probability density of fold changes with the selected ESR-related genes between quartiles (the first quartile to the third quartile) of POLY. [file 1741-7015-10-125-S5.DOC]

**Supplementary Figure 2** The fold changes in expression of the genes that differ between groups and between quartiles of ESR or JC. (A) The probability density of fold changes with the selected ESR-related genes between F1 + Q and F2 of SJIA; (B) The probability density of fold changes with the selected ESR-related genes between quartiles (the 1^st^ quartile to the 3^rd^ quartile) of SJIA; (C) The probability density of fold changes with the selected JC-related genes between Q and F of SJIA; (D) The probability density of fold changes with the selected JC- related genes between quartiles (the 1^st^ quartile to the 3^rd^ quartile) of SJIA; (E) The probability density of fold changes with the selected ESR-related genes between F2 and F1 + Q of POLY; (F) The probability density of fold changes with the selected ESR-related genes between quartiles (the 1^st^ quartile to the 3^rd^ quartile) of POLY.


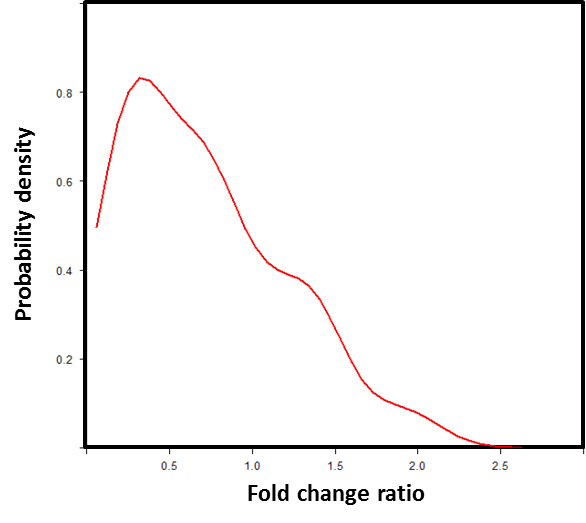

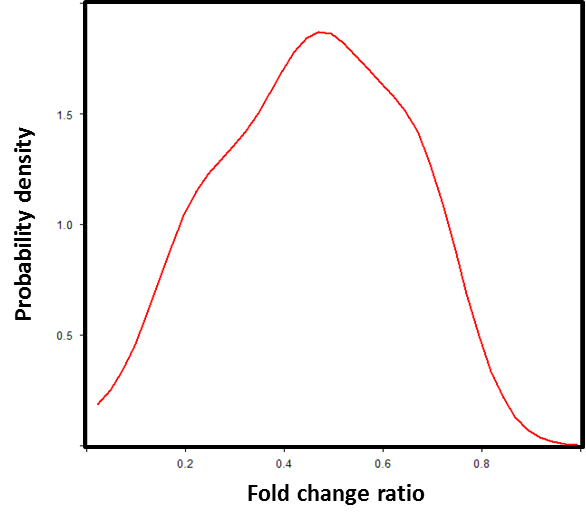

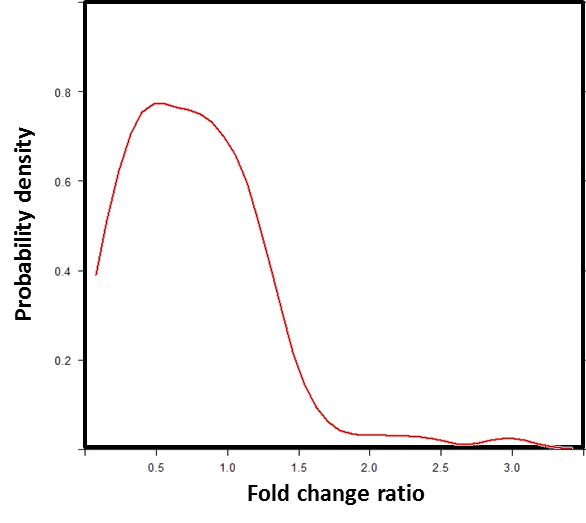


1. (B) (C)


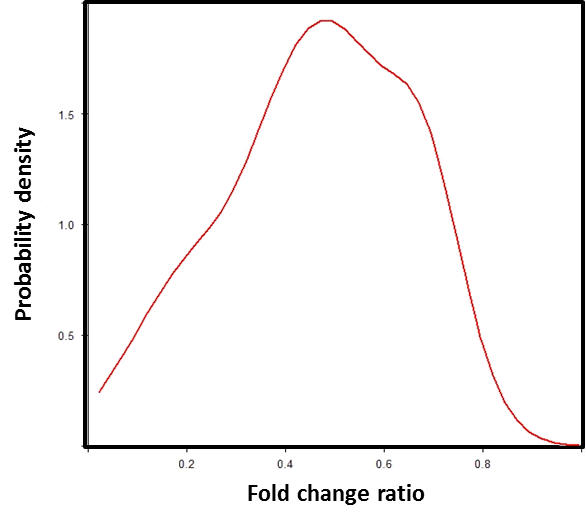

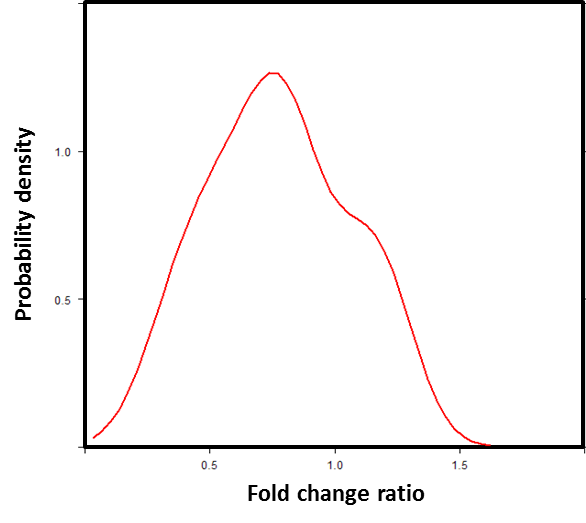

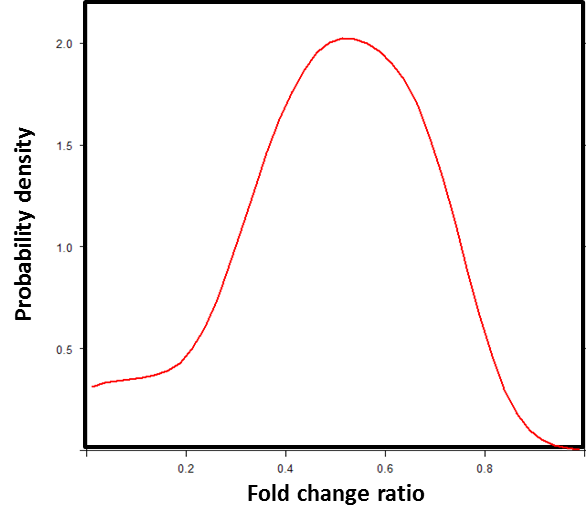


(D) (E) (F)
